# Arterial Thromboembolism Incidence in Japanese Patients With Lung Cancer

**DOI:** 10.1002/cam4.71356

**Published:** 2025-11-20

**Authors:** Kazuki Fukuzawa, Yoshimitsu Shimomura, Ling Zha, Haruka Shida, Manabu Hayama, Tetsuhisa Kitamura, Yoshiharu Horie

**Affiliations:** ^1^ Data Science, Medical Division Osaka Japan; ^2^ Department of Environmental Medicine and Population Science Graduate School of Medicine, Osaka University Osaka Japan; ^3^ Medical Science Department, Late Development Oncology TA Division, Research & Development Tokyo Japan

**Keywords:** administrative claims, epidemiology, healthcare, incidence, lung neoplasms, thrombosis

## Abstract

**Background:**

Patients with lung cancer tend to have a high incidence of arterial thromboembolism (ATE). However, although ATE is a life‐threatening disease, there are insufficient studies evaluating patient characteristics associated with a high risk of ATE in patients with lung cancer. This study aimed to examine the incidence of ATE in Japanese patients with lung cancer and evaluate the incidence of ATE by histological types and treatment patterns to identify high‐risk groups.

**Methods:**

We conducted a retrospective cohort study using the National Health Insurance (NHI), Employees' Health Insurance (EHI), and Later‐Stage Elderly Healthcare System (LSEHS) claims databases from April 1, 2014, to March 31, 2022. The patients were followed up for up to 5 years after lung cancer diagnosis. The cumulative incidence rates of ATE were calculated, and the histological subtype and treatment pattern were analyzed using the Cox proportional hazards model.

**Results:**

Among 6340 patients in EHI, 10,857 patients in NHI, and 13,039 patients in LSEHS, patient characteristics such as the median age and the prevalence of comorbidities were different among the three databases. The cumulative incidence rates at 1 year were 1.7%, 4.5%, and 7.2%, and those at 5 years were 3.5%, 8.0%, and 15.9%. The adjusted hazard ratio for small cell lung cancer compared to non‐small cell lung cancer was 2.84 (95% confidence interval (CI): 1.46–5.54) in EHI, 0.93 (95% CI: 0.65–1.32) in NHI, and 1.11 (95% CI: 0.85–1.46) in LSEHS. The risk of ATE of RT + systemic therapy and systemic therapy only was higher than surgery only, with a statistically significant difference in the three databases.

**Conclusion:**

In any database, the cumulative incidence of ATE increased over the 5 year observation period. Patients who received RT + systemic therapy and those who received systemic therapy only had a higher risk of ATE than those who underwent surgery only.

## 1 Introduction

Lung cancer is one of the most prevalent and lethal malignancies worldwide, and is a leading cause of cancer related mortality [1, 2, 3]. Lung cancer is histologically classified into small cell lung cancer (SCLC) and non‐small cell lung cancer (NSCLC), with adenocarcinoma, squamous cell carcinoma, and large cell carcinoma being the major subtypes of NSCLC [4]. Treatment strategies for lung cancer are determined by several important clinical factors, including disease stage, histological type, and biomarkers [5, 6, 7, 8, 9, 10]. Although there remain many unmet medical needs in the treatment of lung cancer, recent advancements in treatment modalities, such as molecularly targeted therapies and immunotherapies, have extended the survival of lung cancer patients [11, 12, 13]. As the life expectancy of patients with a growing number of lung cancer survivors has improved, management of long term comorbidities has become increasingly important.

Arterial thromboembolism (ATE) among cancer patients has recently been gaining attention because it occurs infrequently (1‐year cumulative incidence rate of ATE: overall: 1.3%–6.5%) but can be a life‐threatening disease [14, 15, 16, 17, 18]. Patients with lung cancer have been reported to have a high incidence of ATE compared to those with other cancers (1‐year cumulative incidence rate of ATE: lung cancer: 1.9%–10.3%). However, while ATE in patients with NSCLC has been evaluated in previous studies, there is insufficient information on patient characteristics, such as histological types or treatment patterns associated with a high risk of ATE in patients with lung cancer [14, 15, 16, 17, 18, 19]. We believe that identifying patient characteristics at high risk for ATE is important information for preventing the onset of ATE. Therefore, this study aimed to examine the incidence of ATE in patients with lung cancer over 5 years using claims databases of three types of medical insurance systems in Japan as the primary objective, evaluate the incidence of ATE by histological types and treatment patterns to identify high‐risk groups among patients with lung cancer as the secondary objectives, and explore the predictors of ATE as the exploratory objective.

## 2 Methods

### 2.1 Study Design and Setting

**FIGURE 1:**
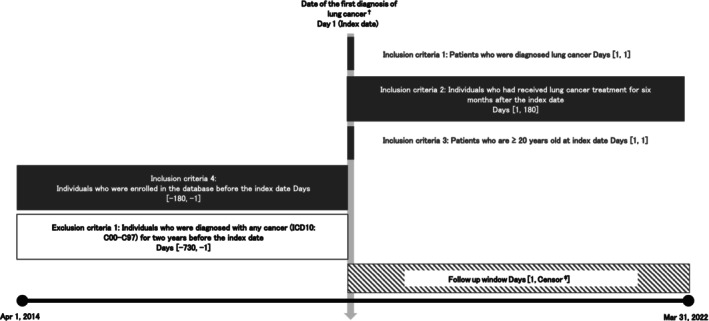
Study design diagram. A graphical depiction of the eligibility criteria and follow‐up windows. †: Lung cancer diagnosis is defined by ICD‐10 code (C340‐C349); ‡: The earliest of the following: First occurrence of ATE, last observable date for the patients, date of second cancer diagnosis, completion of follow‐up (5 years from the index date), or end of the study period (March 31, 2022). The figure was adapted from Schneeweiss et al. [20].

This study was a retrospective, observational cohort study using the Employees' Health Insurance (EHI), National Health Insurance (NHI), and Later‐Stage Elderly Healthcare System (LSEHS) databases from the Japan Medical Data Center database (JMDC) and the DeSC database (DeSC). The study period was from April 1, 2014, to March 31, 2022. Patients with lung cancer who met the eligibility criteria shown in Figure 1 were included in this study. The index date was defined as the date of the first diagnosis of lung cancer. The look‐back period was defined as the 6 months prior to the index date for assessing patient characteristics. The follow‐up period started from the index date and ended on the earliest date of any date of the first occurrence of ATE, the last date when patients could be observed, the date of the second cancer diagnosis, completion of the follow‐up (5 years from the index date), or the end of the study period (March 31, 2022).

### 2.2 Data Sources

We used three types of medical insurance systems: the EHI, NHI, and LSEHS. The Japanese are almost entirely covered by these three types of insurance. The EHI consists of employed workers and their dependents who are relatively young (mainly < 65 years). The NHI consists of self‐employed workers and retirees aged < 75 years. The LSEHS consists of patients who are ≥ 75 years. For some NHI and LSEHS insurers, it is possible to collect information on deaths from the ledgers of insured persons. The EHI data were provided by JMDC Inc., and the NHI and LSEHS data were obtained from DeSC Healthcare Inc. Two databases, the JMDC and DeSC, were targeted from April 1, 2014, to March 31, 2021. The types of data in both databases are claims, including inpatients, outpatients, dispensing receipts, and health checkups received from health insurance societies in Japan, which can be followed by patients even when they receive care in different hospitals, unless they withdraw from their health insurance societies [21, 22].

### 2.3 Target Population

Patients (≥ 20 years old) with a diagnosis of lung cancer from April 1, 2014, to March 31, 2021, received at least one treatment for lung cancer within 6 months on and after the index date, and without any prior documented history of any cancer for 2 years prior to the index date. Lung cancer was classified according to the International Classification of Diseases‐10th Edition (ICD‐10) codes (code C34; Table S1). Lung cancer treatment was defined as the existence of any lung cancer‐related treatment record (surgery, radiation therapy (RT), systemic treatment, or others) within 6 months before and after the index date. Surgery, RT, and other treatments for lung cancer were defined using medical procedure codes. Systemic treatment for lung cancer was selected according to the clinical guidelines for lung cancer treatment [5, 6, 7, 8, 9, 10].

### 2.4 Outcome Definition

ATE was defined as a composite outcome of ischemic stroke and myocardial infarction with hospitalization in accordance with the ICD‐10 codes (codes I63, I21, or I22). Only the first event was considered to estimate the incidence rate.

### 2.5 Statistical Analysis

All analyses were performed for each database (the EHI, NHI, and LSEHS). Baseline characteristics were summarized as mean, standard deviation (SD), median, interquartile ranges, minimum, and maximum for continuous variables, and count and percentage for categorical variables. The cumulative incidence rates of ATE and its 95% confidence interval (CI) were calculated for the overall population and subpopulations stratified by histological subtype (non‐small cell lung cancer (NSCLC), small cell lung cancer (SCLC), and unknown), NSCLC subtype (adenocarcinoma, squamous cell carcinoma, large cell carcinoma, and other), treatment pattern (surgery only, surgery + systemic therapy, RT only, RT + systemic therapy, systemic therapy only, and other), and systemic therapy subtype (chemotherapy, molecularly targeted therapy, immune checkpoint inhibitor, multiple therapy, and other therapy) using the Nelson‐Aalen estimator. Histological subtype was defined based on disease names and disease codes associated with the ICD‐10 code C34. The definitions of histological subtype, algorithms for treatment pattern, and algorithms for systemic therapy subtype are shown in Tables S1, S2, and S3. The adjusted Hazard Ratio (aHR) and its corresponding 95% confidence interval (CI) were calculated using the Breslow method to assess the difference in ATE risk between histological subtype and treatment pattern and to explore the risk factors for ATE in patients with lung cancer [23]. The Cox proportional hazards model included sex, age at cancer diagnosis, histological subtype, brain metastasis (disease code: 1983019), treatment pattern, comorbidities (heart failure, hypertension, atrial fibrillation/flutter, coronary artery disease, venous thromboembolism, chronic kidney disease, diabetes mellitus, dyslipidemia, chronic obstructive pulmonary disease, and dementia), and a history of ATE as covariates (Table S4). We calculated the aHR and its corresponding 95% CI in patients without a history of ATE as a sensitivity analysis. The cumulative incidence rate of ATE and its 95% CI, considering death as a competing risk in the NHI and LSEHS, was calculated using the Breslow method. If the reason for the withdrawal from insurance was death, the patient was considered dead. The date of death was defined as the year and month of insurance withdrawal. Statistical analyses were performed using SAS 9.4. The two‐sided level of statistical significance was set at *p* < 0.05.

### 2.6 Ethics

This study complied with the principles of the Declaration of Helsinki and was approved by the Specified Non‐profit Corporation MINDS (approval number MINS‐REC‐240221) and the Institutional Review Board of Osaka University Hospital (approval number 24264).

## 3 Results

### 3.1 Study Population

From April 1, 2014, to March 31, 2021, 23,944 patients in the EHI, 42,438 patients in the NHI and 106,915 patients in the LSEHS were diagnosed with lung cancer, of whom 6340 (26.5%) were fulfilled the eligibility criteria in the EHI, 10,857 (25.6%) in the NHI, and 13,039 (12.2%) in the LSEHS (Figure S1). Table 1 shows the patient demographics and clinical characteristics in each database. The median age was 60.2, 68.8, and 79.6 years for the EHI, NHI, and LSEHS. The proportion of histological classification of lung cancer was similar among the three databases, NSCLC (43.7%–46.6%), SCLC (6.3%–9.4%), and unknown (44.8%–48.4%). The EHI had the highest percentage of patients treated with surgery only (33.2%), whereas the NHI and LSEHS had the highest percentages of patients treated with systemic therapy (32.5% and 33.9%, respectively). The RT only (15.1%) was conducted in the LSEHS, which was higher than in the others. The proportion of patients with comorbidities, medication history, and history of ATE in the LSEHS was the highest among the three databases.

**TABLE 1:** Demographics and clinical characteristics.

	EHI	NHI	LSEHS
Patients, *n*	6340	10,857	13,039
Age, year^a^			
mean [SD]	59.1 [9.0]	67.4 [5.6]	80.5 [4.3]
median [Q1–Q3]	60.2 [53.5–65.5]	68.8 [65.8–71.0]	79.6 [77.4–83.0]
Age > = 65, *n* (%)^a^	1741 (27.5)	8598 (79.2)	13,039 (100.0)
Male, *n* (%)^a^	4341 (68.5)	7368 (67.9)	8120 (62.3)
Histological subtypes of lung cancer, *n* (%)^b^			
NSCLC	2954 (46.6)	4969 (45.8)	5701 (43.7)
Adenocarcinoma	1722 (27.2)	2588 (23.8)	2824 (21.7)
Squamous cell carcinoma	344 (5.4)	1031 (9.5)	1214 (9.3)
Large cell carcinoma	28 (0.4)	50 (0.5)	24 (0.2)
Other	860 (13.6)	1300 (12.0)	1639 (12.6)
SCLC	399 (6.3)	1019 (9.4)	1026 (7.9)
Unknown	2987 (47.1)	4869 (44.8)	6312 (48.4)
Brain metastasis, *n* (%)^c^	719 (11.3)	1700 (15.7)	1344 (10.3)
Treatment types (%)^c^			
Surgery only	2106 (33.2)	2615 (24.1)	3825 (29.3)
Surgery + Systemic therapy	1259 (19.9)	1371 (12.6)	910 (7.0)
Radiation therapy only	147 (2.3)	558 (5.1)	1975 (15.1)
Radiation therapy + Systemic therapy	1201 (18.9)	2582 (23.8)	1795 (13.8)
Systemic therapy only	1494 (23.6)	3526 (32.5)	4414 (33.9)
Chemotherapy	570 (9.0)	1401 (12.9)	1642 (12.6)
Molecularly targeted therapy	313 (4.9)	572 (5.3)	936 (7.2)
Immune checkpoint inhibitor	63 (1.0)	197 (1.8)	372 (2.9)
Multiple therapy	497 (7.8)	1198 (11.0)	774 (5.9)
Other therapy	51 (0.8)	158 (1.5)	690 (5.3)
Others	133 (2.1)	205 (1.9)	120 (0.9)
Comorbidities, *n* (%)^¶^			
Heart Failure	426 (6.7)	1121 (10.3)	3479 (26.7)
Hypertension	2133 (33.6)	4934 (45.4)	9149 (70.2)
Atrial Fibrillation/flutter	141 (2.2)	412 (3.8)	1181 (9.1)
Coronary artery disease	468 (7.4)	1259 (11.6)	3162 (24.3)
Venous thromboembolism	19 (0.3)	30 (0.3)	64 (0.5)
Chronic kidney disease	79 (1.2)	255 (2.3)	812 (6.2)
Diabetes mellitus	1164 (18.4)	3108 (28.6)	5089 (39.0)
Dyslipidemia	1835 (28.9)	4028 (37.1)	7086 (54.3)
Chronic obstructive pulmonary disease	416 (6.6)	963 (8.9)	1794 (13.8)
Dementia	10 (0.2)	72 (0.7)	628 (4.8)
History of ATE, *n* (%)^¶^	193 (3.0)	739 (6.8)	1883 (14.4)

Abbreviations: ATE, arterial thromboembolism; EHI, Employees' Health Insurance; LSEHS, Later‐Stage Elderly Healthcare System; NHI, National Health Insurance; NSCLC, non‐small cell lung cancer; SCLC, small cell lung cancer; SD, standard deviation.

^a^ Index date.

^b^ Within 3 months on and after the index date.

^c^ Within 6 months of and after the index date.

^¶^ Look‐back period (6‐month period prior to the index date).

### 3.2 Cumulative Incidence Rate of ATE in the Overall Population

**FIGURE 2:**
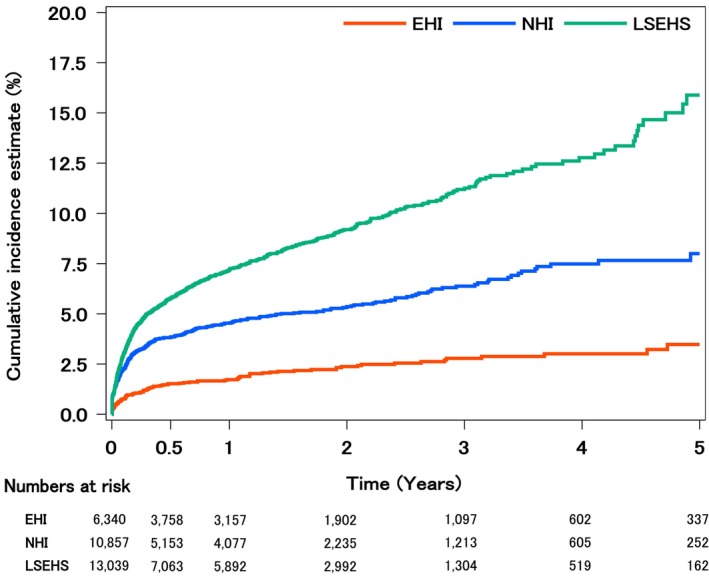
Cumulative incidence of ATE. The vertical axis indicates the cumulative incidence rate, and the horizontal axis indicates the number of years after lung cancer diagnosis. The cumulative incidence of ATE is illustrated by data type (the EHI, NHI, and LSEHS). ATE = Arterial thromboembolism; EHI = Employees' Health Insurance; NHI = National Health Insurance; LSEHS = Later‐Stage Elderly Healthcare System.

The cumulative incidence rates of ATE within 1 year of lung cancer diagnosis were 1.7% in the EHI, 4.5% in the NHI, and 7.2% in the LSEHS, and the cumulative incidence rates of ATE within 5 years from lung cancer diagnosis were 3.5% in the EHI, 8.0% in the NHI, and 15.9% in the LSEHS (Table 2 and Figure 2). In all three databases, the incidence within 1 year after cancer diagnosis accounted for approximately half of the total incidence during a 5‐year follow‐up period. Analyses of the NHI and LSEHS, which considered death as a competing risk, showed a similar trend to the primary analysis results.

**TABLE 2:** Cumulative incidence rate (95% CI^a^) of ATE by years after lung cancer diagnosis.

	3‐month	6‐month	1‐year	2‐year	3‐year	4‐year	5‐year
EHI	1.10 [0.85–1.43]	1.52 [1.21–1.91]	1.72 [1.39–2.14]	2.37 [1.93–2.91]	2.78 [2.25–3.44]	3.01 [2.41–3.75]	3.47 [2.66–4.53]
NHI	3.24 [2.88–3.64]	3.82 [3.42–4.27]	4.54 [4.08–5.04]	5.36 [4.81–5.96]	6.38 [5.68–7.16]	7.48 [6.56–8.53]	8.00 [6.85–9.32]
LSEHS	4.64 [4.25–5.06]	5.77 [5.33–6.25]	7.20 [6.68–7.75]	9.19 [8.55–9.88]	11.26 [10.41–12.17]	12.77 [11.65–14.00]	15.88 [13.81–18.23]

Abbreviation: CI, confidence interval. Other abbreviations as in Table 1.

^a^ Nelson–Aalen confidence interval.

### 3.3 Cumulative Incidence Rate and aHR of ATE in the Subgroups

**FIGURE 3:**
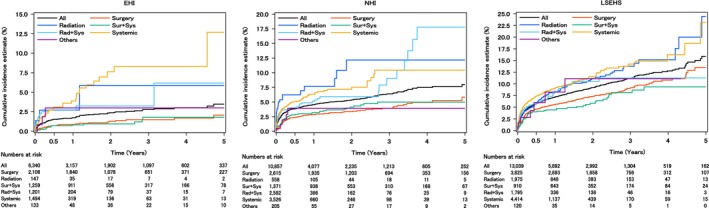
Cumulative incidence of ATE by treatment pattern. The vertical axis indicates the cumulative incidence rate, and the horizontal axis indicates the number of years after lung cancer diagnosis. The cumulative incidence of ATE is illustrated by the treatment pattern of lung cancer in the EHI, NHI, and LSEHS. Radiation = radiation therapy only; Rad+Sys = radiation therapy + systemic therapy; Sur + Sys = surgery + systemic therapy; systemic = systemic therapy only; Other abbreviations as in Figure 2.

**FIGURE 4:**
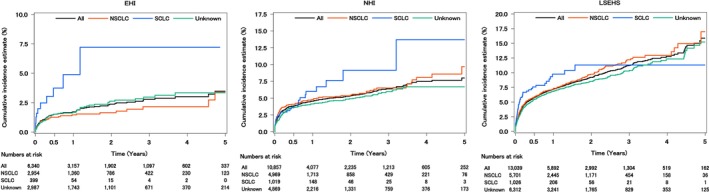
Cumulative incidence of ATE by histological subtype. The vertical axis indicates the cumulative incidence rate, and the horizontal axis indicates the number of years after lung cancer diagnosis. The cumulative incidence of ATE is illustrated by the histological subtype in the EHI, NHI, and LSEHS. NSCLC = non‐small cell lung cancer; SCLC = small cell lung cancer. Other abbreviations are as in Figure 2.

Analysis stratified by treatment pattern shows that the cumulative incidence rates for radiotherapy only (EHI: 1‐year: 2.7%, 5‐year: 5.9%; NHI: 1‐year: 7.7%, 5‐year: 12.2%; LSEHS: 1‐year: 8.3%, 5‐year: 24.4%), radiotherapy + systemic therapy (EHI: 1‐year: 2.7%, 5‐year: 6.2%; NHI: 1‐year: 5.1%, 5‐year: 17.8%; LSEHS: 1‐year: 8.4%, 5‐year: 11.3%), and systemic therapy only (EHI: 1‐year: 3.9%, 5‐year: 12.7%; NHI: 1‐year: 6.3%, 5‐year: 10.4%; LSEHS: 1‐year: 9.1%, 5‐year: 23.2%) tended to be higher than that for the overall population over all time periods in any databases (Figure 3 and Table S5). The aHRs of ATE following lung cancer diagnosis according to treatment patterns are shown in Table 3. The risk of ATE of RT + systemic therapy (EHI: aHR: 3.59 [95% CI: 1.95–6.64]; NHI: aHR: 2.06 [95% CI: 1.49–2.84]; LSEHS: aHR: 1.46 [95% CI: 1.13–1.89]) and systemic therapy only (EHI: aHR: 5.34 [95% CI: 3.11–9.17]; NHI: aHR: 2.46 [95% CI: 1.85–3.29]; LSEHS: aHR: 1.75 [95% CI: 1.47–2.09]) were higher than surgery only with a statistically significance in the three databases. There was no significant difference between A and B. No systemic therapy subtype showed a statistically significant difference in the incidence of ATE compared to chemotherapy in any database (Table 3). In patients without a history of ATE, the adjusted hazard ratios (HRs) showed a similar trend to the main analysis in any database (Table S7). Analysis stratified by histological subtype shows that the cumulative incidence rate for SCLC (EHI: 1‐year: 4.9%, 5‐year: 7.2%; NHI: 1‐year: 6.0%, 5‐year: 13.7%; LSEHS: 1‐year: 9.8%, 5‐year: 11.3%) tended to be higher than that of NSCLC (EHI: 1‐year: 1.5%, 5‐year: 3.4%; NHI: 1‐year: 4.8%, 5‐year: 9.7%; LSEHS: 1‐year: 7.2%, 5‐year: 17.0%) in any databases (Figure 4 and Table S5). The aHRs for SCLC compared to NSCLC were 2.84 (95% CI: 1.46–5.54), 0.93 (95% CI: 0.65–1.32), 1.11 (95% CI: 0.85–1.46) in the EHI, NHI, and LSEHS (Table 3). Analysis stratified by NSCLC subtype showed that the 1‐year and 5‐years cumulative incidence rates for adenocarcinoma were 0.9% and 3.1% in the EHI, 4.1% and 8.4% in the NHI, and 7.3% and 16.6% in the LSEHS, respectively, and those for squamous cell carcinoma were 2.3% and 6.9% in the EHI, 6.0% and 19.8% in the NHI, and 8.6% and 21.9% in the LSEHS, respectively. In large cell carcinoma, no patients in the EHI and NHI experienced ATE, and 9.8% of patients in the LSEHS experienced ATE within 1 year of lung cancer diagnosis (Table S5). No NSCLC subtype showed a statistically significant difference in the incidence of ATE compared to adenocarcinoma in any database in any database (Table 3).

**TABLE 3:** Adjusted hazard ratio (95% CI^b^) of ATE during the 5‐year period following cancer diagnosis.

	Patients, *n*	Event, *n* (%)	Person‐years	Mean of Follow‐up Period (year)	Adjusted hazard ratio^a^	95% CI^b^
EHI						
Histological subtype						
NSCLC	2954	39 (1.3)	3800	1.29	Reference	
SCLC	399	12 (3.0)	179	0.45	2.84	[1.46–5.54]
Unknown	2987	59 (2.0)	5098	1.71	1.18	[0.78–1.77]
NSCLC subtype						
Adenocarcinoma	1722	17 (1.0)	2407	1.40	Reference	
Squamous cell carcinoma	344	7 (2.0)	358	1.04	1.68	[0.68–4.19]
Large cell carcinoma	28	0 (0.0)	25	0.89	—	
Others	860	15 (1.7)	1010	1.17	1.64	[0.80–3.33]
Treatment Pattern						
Surgery only	2106	26 (1.2)	4783	2.27	Reference	
Surgery + Systemic therapy	1259	13 (1.0)	2529	2.01	0.99	[0.50–1.93]
RT only	147	4 (2.7)	97	0.66	1.94	[0.60–6.22]
RT + Systemic therapy	1201	24 (2.0)	600	0.50	3.59	[1.95–6.64]
Systemic therapy only	1494	40 (2.7)	898	0.60	5.34	[3.11–9.17]
Others	133	3 (2.3)	172	1.29	3.32	[0.99–11.13]
Systemic therapy subtype						
Chemotherapy	570	40 (2.7)	372	0.65	Reference	
Molecularly targeted therapy	313	16 (2.8)	203	0.65	1.15	[0.40–3.30]
Immune checkpoint inhibitor	63	6 (1.9)	58	0.92	1.42	[0.31–6.52]
Multiple therapy	497	2 (3.2)	238	0.48	1.49	[0.69–3.22]
Other therapy	51	14 (2.8)	26	0.51	1.72	[0.32–9.41]
NHI						
Histological subtype						
NSCLC	4969	193 (3.9)	4648	0.94	Reference	
SCLC	1019	37 (3.6)	505	0.50	0.93	[0.65–1.32]
Unknown	4869	179 (3.7)	6306	1.30	0.77	[0.63–0.94]
NSCLC subtype						
Adenocarcinoma	2588	94 (3.6)	2618	1.01	Reference	
Squamous cell carcinoma	1031	50 (4.8)	890	0.86	1.06	[0.75–1.52]
Large cell carcinoma	50	0 (0.0)	37	0.74	—	
Others	1300	49 (3.8)	1103	0.85	1.00	[0.70–1.41]
Treatment Pattern						
Surgery only	2615	89 (3.4)	5353	2.05	Reference	
Surgery + Systemic therapy	1371	50 (3.6)	2570	1.87	1.18	[0.83–1.67]
RT only	558	33 (5.9)	293	0.53	2.91	[1.92–4.39]
RT + Systemic therapy	2582	85 (3.3)	1167	0.45	2.06	[1.49–2.84]
Systemic therapy only	3526	147 (4.2)	1902	0.54	2.46	[1.85–3.29]
Others	205	5 (2.4)	174	0.85	1.25	[0.50–3.08]
Systemic therapy subtype						
Chemotherapy	1401	55 (3.9)	725	0.52	Reference	
Molecularly targeted therapy	572	16 (2.8)	339	0.59	0.85	[0.47–1.56]
Immune checkpoint inhibitor	197	11 (5.6)	123	0.62	1.38	[0.70–2.71]
Multiple therapy	1198	55 (4.6)	650	0.54	1.10	[0.74–1.64]
Other therapy	158	10 (6.3)	65	0.41	2.16	[1.06–4.42]
LSEHS						
Histological subtype						
NSCLC	5701	369 (6.5)	5991	1.05	Reference	
SCLC	1026	63 (6.1)	566	0.55	1.11	[0.85–1.46]
Unknown	6312	416 (6.6)	8350	1.32	0.84	[0.73–0.97]
NSCLC subtype						
Adenocarcinoma	2824	179 (6.3)	3139	1.11	Reference	
Squamous cell carcinoma	1214	105 (8.6)	1242	1.02	1.18	[0.92–1.53]
Large cell carcinoma	24	2 (8.3)	28	1.17	1.24	[0.31–5.04]
Others	1639	83 (5.1)	1582	0.97	0.79	[0.61–1.03]
Treatment Pattern						
Surgery only	3825	273 (7.1)	7251	1.90	Reference	
Surgery + Systemic therapy	910	52 (5.7)	1632	1.79	0.93	[0.69–1.25]
RT only	1975	146 (7.4)	2075	1.05	1.24	[1.00–1.52]
RT + Systemic therapy	1795	84 (4.7)	915	0.51	1.46	[1.13–1.89]
Systemic therapy only	4414	286 (6.5)	2942	0.67	1.75	[1.47–2.09]
Others	120	7 (5.8)	93	0.78	1.67	[0.79–3.55]
Systemic therapy subtype						
Chemotherapy	1642	119 (7.2)	858	0.52	Reference	
Molecularly targeted therapy	936	42 (4.5)	727	0.78	0.67	[0.45–1.00]
Immune checkpoint inhibitor	372	33 (8.9)	305	0.82	0.89	[0.59–1.33]
Multiple therapy	774	52 (6.7)	420	0.54	0.93	[0.66–1.30]
Other therapy	690	40 (5.8)	631	0.91	0.72	[0.47–1.09]

Abbreviations: CI, confidence interval; NSCLC, non‐small cell lung cancer; SCLC, small cell lung cancer; RT, Radiation therapy. Other abbreviations are shown in Table 1.

^a^ Adjusted for sex, age at cancer diagnosis, histological subtype, brain metastasis, treatment pattern, comorbidities (heart failure, hypertension, atrial fibrillation/flutter, coronary artery disease, venous thromboembolism, chronic kidney disease, diabetes mellitus, dyslipidemia, chronic obstructive pulmonary disease, and dementia), and history of ATE.

^b^ Wald's confidence interval.

### 3.4 Predictive Factors of ATE

Table 4 shows the factors associated with the ATE. Male patients were more likely to develop ATE than female patients, and patients with a history of ATE were more likely to develop ATE after a lung cancer diagnosis. Age was identified as a predictive factor for ATE in the NHI. In the EHI, dyslipidemia, and in the LSEHS, some comorbidities (atrial fibrillation/flutter, coronary artery disease, diabetes mellitus, and dementia) were predictive of ATE. In the NHI, comorbidities were not predictive of ATE.

**TABLE 4:** Predictive factors of ATE among patients with lung cancer.

	Adjusted hazard ratio (95% CI^a^)		
	EHI	NHI	LSEHS
Histological subtype (Reference: NSCLC)			
SCLC	2.84 [1.46–5.54]	0.93 [0.65–1.32]	1.11 [0.85–1.46]
Unknown	1.18 [0.78–1.77]	0.77 [0.63–0.94]	0.84 [0.73–0.97]
Sex (Reference: Male)	0.44 [0.26–0.74]	0.60 [0.47–0.78]	0.72 [0.61–0.84]
Age	1.01 [0.98–1.04]	1.03 [1.01–1.06]	1.00 [0.98–1.02]
Heart failure	1.31 [0.71–2.41]	0.99 [0.74–1.33]	0.93 [0.79–1.09]
Hypertension	1.02 [0.66–1.59]	1.23 [0.98–1.55]	1.19 [0.99–1.43]
Atrial fibrillation/flutter	0.82 [0.33–2.03]	1.13 [0.77–1.65]	1.38 [1.13–1.69]
Coronary artery disease	0.65 [0.36–1.18]	1.21 [0.93–1.58]	1.22 [1.05–1.43]
Venous thromboembolism	—	—	0.33 [0.08–1.30]
Chronic kidney disease	0.49 [0.12–2.09]	0.81 [0.45–1.45]	1.07 [0.84–1.35]
Diabetes mellitus	1.32 [0.84–2.08]	1.09 [0.88–1.36]	1.31 [1.13–1.51]
Dyslipidemia	1.91 [1.19–3.06]	0.99 [0.79–1.24]	0.91 [0.78–1.06]
COPD	1.02 [0.54–1.95]	1.08 [0.80–1.44]	0.90 [0.74–1.08]
Dementia	3.74 [0.83–16.88]	1.50 [0.70–3.19]	1.32 [1.01–1.72]
History of ATE	11.18 [6.88–18.17]	7.47 [5.96–9.36]	5.09 [4.41–5.87]

Abbreviations: ATE, arterial thromboembolism; CI, confidence interval; NSCLC, non‐small‐cell lung cancer; SCLC, small‐cell lung cancer; COPD, chronic obstructive pulmonary disease; Other abbreviations as in Table 1.

^a^ Wald confidence interval.

## 4 Discussion and Conclusion

This study comprehensively evaluated ATE in patients with lung cancer over a 5‐year follow‐up period from cancer diagnosis using claims databases of all types of medical insurance systems in Japan (the EHI, NHI, and LSEHS). The novelty of this study lies in the evaluation of ATE using databases that nearly represent the entire population in Japan, and the assessment by histological subtype and treatment pattern to identify high‐risk groups. Regardless of histological subtype or treatment pattern, the incidence rate tended to be higher in EHI, followed by NHI and LSEHS. In the EHI, which had the lowest median age among the three databases, patients with SCLC were more likely to develop ATE than those with NSCLC, while there was no difference in the NHI and LSEHS. The NSCLC subtype had no effect on ATE. Patients who received RT + systemic therapy and those who received systemic therapy only had a higher risk of ATE than those who underwent surgery only in the three claims databases. The systemic therapy subtype had no effect on ATE. These results provide important information for the management of ATE in patients with lung cancer.

The incidence of ATE within 1 year after cancer diagnosis varied across three claims databases (1.7% in the EHI, 4.5% in the NHI, and 7.2% in the LSEHS), which suggests that the age and the prevalence of comorbidities such as atrial fibrillation/flutter or chronic kidney disease among patients included in the databases may influence the occurrence of ATE. These results were generally within the range of incidence rates reported in previous studies (1.9%–10.3%) [14, 15, 16, 17, 18]. The lower incidence of ATE in the EHI compared to that in previous studies may be because the EHI mainly comprises patients aged 65 years or younger. The incidence of ATE within 5 years of cancer diagnosis (3.5% in the EHI, 8.0% in the NHI, and 15.9% in the LSEHS) tended to be higher than that reported in a retrospective study using the Osaka Cancer Registry (3.1%) [15]. Those differences might depend on the differences in patient characteristics between the studies, such as the median of age (70 vs. 60.2–79.6), the high prevalence of hypertension (16.7% vs. 33.6%–70.2%), heart failure (2.3% vs. 6.7%–26.7%), and previous ATE (1.4% vs. 3.0%–14.4%), and the high rate of advanced cancer.

Analysis by treatment pattern revealed an increased risk of ATE in patients who received RT + systemic therapy and those who received systemic therapy only, compared to patients who underwent surgery only in the three claims databases. In clinical practice, treatment decisions are influenced by factors such as age, comorbidities, or spirometry. However, according to the Japanese and international clinical guidelines for lung cancer, surgery is typically performed for patients with early‐stage cancer, while systemic therapy is primarily used for advanced cancer [6, 7, 8, 9, 10]. As previous studies have reported that the risk of ATE increases as cancer progresses, in our study, ATE might also be influenced by cancer progression [14, 15, 16].

It is also possible that the treatment modalities themselves influence the risk of ATE. Systemic therapies, including chemotherapy, are known to have cardiotoxic effects, and the platinum agents and taxanes used in lung cancer treatment have been reported to be associated with thromboembolic events [24]. Additionally, among molecular targeted therapies, angiogenesis inhibitors have a known mechanism of action that promotes thromboembolism formation, and their association with thromboembolic events has been reported, suggesting that these drugs may influence the occurrence of ATE [24]. Arterial occlusion and stenosis due to radiation have been reported during RT, indicating that RT may increase the risk of ATE [25]. Although surgery is known to increase the risk of postoperative thromboembolic events, the patient cohort undergoing surgery typically includes patients who have passed preoperative cardiac and pulmonary function tests, implying a potentially lower risk of ATE in this group. Although the possibility that the treatment methods themselves influenced the risk of ATE cannot be ruled out, this study did not evaluate the specific treatment modalities or the time to treatment initiation from cancer diagnosis in detail. Therefore, further research is required to assess the effect of treatment methods on ATE.

The risk of ATE stratified by histological subtype differed among the three claims databases. The EHI results showed that the risk of ATE in patients with SCLC was higher than that in patients with NSCLC. This could be due to differences in malignancy. SCLC is a highly malignant tumor characterized by rapid growth and early lymph node and distant metastases, and is often already at an advanced stage at the time of diagnosis [26]. The proportion of patients receiving systemic therapy was higher among patients with SCLC than among those with NSCLC. (43.1% and 28.5%, respectively; Table S6). Thus, patients with SCLC may have a higher proportion of advanced lung cancer than those with NSCLC, which may have led to a higher incidence of ATE. In contrast, the NHI and LSEHS showed no difference in ATE risk according to histological subtype. These results may be due to differences in patient backgrounds among the databases, such as median age and the proportion of patients with comorbidities, which influence the risk of ATE. For example, in the LSEHS, which consists of patients aged 75 years and older, the effect of age might have been more significant than the effect of the histological subtype; hence, ATE may have occurred regardless of the histological subtype. Additionally, no difference in the ATE risk of the NSCLC subtype was found for squamous cell carcinoma and large cell carcinoma compared to adenocarcinoma across the three databases. Based on our results, not only is the management of ATE important in older patients with lung cancer who have a higher incidence of ATE, but close attention to ATE risk may also be necessary when treating younger patients with SCLC.

Analysis of the predictors of ATE showed that males were more likely to develop ATE than females, and patients with a history of ATE were more likely to develop ATE after a diagnosis of lung cancer. These results reinforce the findings of the previous study [16].

This study focused on patients with lung cancer undergoing treatment and investigated the association between lung cancer and ATE; therefore, the impact of lung cancer itself on ATE was not assessed. Additionally, approximately 40% of patients with lung cancer could not be classified as having NSCLC or SCLC based on the ICD‐10 code. If the histological type of patients with lung cancer could be classified, we would be able to assess a more precise association between the histological subtype and the incidence of ATE. Regarding the limitations related to claims databases, the lack of clinical data (e.g., clinical test results, cancer staging, family history of ATE, and smoking information) or the intent of treatment (e.g., curative and palliative) could potentially influence the results. In particular, the absence of smoking information, a paramount confounding factor for lung cancer and ATE, from claims databases is an important limitation. Moreover, while the NHI and LSEHS include an evaluation of competing risks of mortality, the EHI has partially missing mortality data, which prevents the evaluation of the impact of competing mortality risks when estimating the primary outcome of cumulative incidence. In addition, because this study was conducted using secondary medical data, eliminating the influence of unmeasured confounding factors was challenging.

In conclusion, in any database, the cumulative incidence of ATE increased over the 5‐year observation period, and patients who received RT + systemic therapy and those who received systemic therapy only had a higher risk of ATE than those who underwent surgery only. These findings provide valuable information for managing the risk of ATE in patients with lung cancer.

## Author Contributions

**Kazuki Fukuzawa:** conceptualization, data curation, formal analysis, investigation, methodology, software, visualization, writing – original draft, writing – review and editing. **Yoshimitsu Shimomura:** conceptualization, formal analysis, methodology, software, validation, writing – review and editing. **Ling Zha:** conceptualization, methodology, writing – review and editing. **Haruka Shida:** conceptualization, data curation, formal analysis, investigation, methodology, project administration, software, validation, writing – review and editing. **Manabu Hayama:** conceptualization, methodology, writing – review and editing. **Tetsuhisa Kitamura:** conceptualization, methodology, supervision, writing – review and editing. **Yoshiharu Horie:** conceptualization, methodology, resources, supervision, funding acquisition, writing – review and editing.

## Ethics Statement

Approval of the research protocol by an Institutional Review Board: This study was approved by the Specified Non‐profit Corporation MINDS (approval number MINS‐REC‐240221) and the Institutional Review Board of Osaka University Hospital (approval number 24264).

## Consent

The authors have nothing to report.

## Conflicts of Interest

Kazuki Fukuzawa, Haruka Shida, Manabu Hayama, and Yoshiharu Horie are employees of AstraZeneca. Tetsuhisa Kitamura received research funding from AstraZeneca. Yoshimitsu Shimomura and Ling Zha have nothing to disclose.

## Supporting information


**Data S1:** cam471356‐sup‐0001‐supinfo.docx.

## Data Availability

The JMDC and DeSC database are not contractually authorized for public access. Data archiving is not mandated, but data will be made available at reasonable request.

## References

[1] F. Bray , M. Laversanne , H. Sung , et al., “Global Cancer Statistics 2022: GLOBOCAN Estimates of Incidence and Mortality Worldwide for 36 Cancers in 185 Countries,” CA: A Cancer Journal for Clinicians 74, no. 3 (2024): 229–263, 10.3322/caac.21834. 38572751

[2] L. A. Torre , F. Bray , R. L. Siegel , J. Ferlay , J. Lortet‐Tieulent , and A. Jemal , “Global Cancer Statistics, 2012,” CA: A Cancer Journal for Clinicians 65, no. 2 (2015): 87–108, 10.3322/caac.21262. 25651787

[3] Cancer statistics (Lung) , “National Cancer Center Japan,” https://ganjoho.jp/reg_stat/statistics/stat/cancer/12_lung.html.

[4] Y. Zhang , S. Vaccarella , E. Morgan , et al., “Global Variations in Lung Cancer Incidence by Histological Subtype in 2020: A Population‐Based Study,” Lancet Oncology 24, no. 11 (2023): 1206–1218, 10.1016/S1470-2045(23)00444-8. 37837979

[5] H. Horinouchi , M. Kusumoto , Y. Yatabe , K. Aokage , S. i. Watanabe , and S. Ishikura , “Lung Cancer in Japan,” Journal of Thoracic Oncology 17, no. 3 (2022): 353–361, 10.1016/j.jtho.2021.11.020. 35216731

[6] The Japan Lung Cancer Society , Guidelines for Lung Cancer Treatment ‐ Including Malignant Pleural Mesothelioma and Thymic Tumors ‐ 2024, 8th ed. (Kanehara & Co., Ltd., 2024).

[7] A. M. C. Dingemans , M. Früh , A. Ardizzoni , et al., “Small‐Cell Lung Cancer: ESMO Clinical Practice Guidelines for Diagnosis, Treatment and Follow‐Up,” Annals of Oncology 32, no. 7 (2021): 839–853, 10.1016/j.annonc.2021.03.207. 33864941 PMC9464246

[8] P. E. Postmus , K. M. Kerr , M. Oudkerk , et al., “Early and Locally Advanced Non‐Small‐Cell Lung Cancer (NSCLC): ESMO Clinical Practice Guidelines for Diagnosis, Treatment and Follow‐Up†,” Annals of Oncology 28 (2017): iv1–iv21, 10.1093/annonc/mdx222. 28881918

[9] L. E. Hendriks , K. M. Kerr , J. Menis , et al., “Non‐Oncogene‐Addicted Metastatic Non‐Small‐Cell Lung Cancer: ESMO Clinical Practice Guideline for Diagnosis, Treatment and Follow‐Up,” Annals of Oncology 34, no. 4 (2023): 358–376, 10.1016/j.annonc.2022.12.013. 36669645

[10] L. E. Hendriks , K. M. Kerr , J. Menis , et al., “Oncogene‐Addicted Metastatic Non‐Small‐Cell Lung Cancer: ESMO Clinical Practice Guideline for Diagnosis, Treatment and Follow‐Up☆,” Annals of Oncology 34, no. 4 (2023): 339–357, 10.1016/j.annonc.2022.12.009. 36872130

[11] S. Ramalingam Suresh , J. Vansteenkiste , D. Planchard , et al., “Overall Survival With Osimertinib in Untreated, EGFR‐Mutated Advanced NSCLC,” New England Journal of Medicine 382, no. 1 (2020): 41–50, 10.1056/NEJMoa1913662. 31751012

[12] M. Reck , D. Rodríguez‐Abreu , A. G. Robinson , et al., “Five‐Year Outcomes With Pembrolizumab Versus Chemotherapy for Metastatic Non–Small‐Cell Lung Cancer With PD‐L1 Tumor Proportion Score ≥ 50%,” Journal of Clinical Oncology 39, no. 21 (2021): 2339–2349, 10.1200/JCO.21.00174. 33872070 PMC8280089

[13] L. Paz‐Ares , Y. Chen , N. Reinmuth , et al., “Durvalumab, With or Without Tremelimumab, Plus Platinum‐Etoposide in First‐Line Treatment of Extensive‐Stage Small‐Cell Lung Cancer: 3‐Year Overall Survival Update From CASPIAN,” ESMO Open 7, no. 2 (2022): 100408, 10.1016/j.esmoop.2022.100408. 35279527 PMC9161394

[14] B. B. Navi , A. S. Reiner , H. Kamel , et al., “Risk of Arterial Thromboembolism in Patients With Cancer,” Journal of the American College of Cardiology 70, no. 8 (2017): 926–938, 10.1016/j.jacc.2017.06.047. 28818202 PMC5667567

[15] Y. Gon , T. Morishima , T. Kawano , et al., “Arterial Thromboembolism in Japanese Patients With Cancer: Incidence, Predictors, and Survival Impact,” JACC Cardio Oncology 6, no. 2 (2024): 283–297, 10.1016/j.jaccao.2024.01.006. PMC11103032 38774004

[16] F. I. Mulder , E. Horváth–Puhó , N. van Es , et al., “Arterial Thromboembolism in Cancer Patients: A Danish Population–Based Cohort Study,” JACC: CardioOncology 3, no. 2 (2021): 205–218, 10.1016/j.jaccao.2021.02.007. 34396325 PMC8352038

[17] E. Grilz , O. Königsbrügge , F. Posch , et al., “Frequency, Risk Factors, and Impact on Mortality of Arterial Thromboembolism in Patients With Cancer,” Haematologica 103, no. 9 (2018): 1549–1556, 10.3324/haematol.2018.192419. 29794142 PMC6119137

[18] S. Feldman , D. Gupta , B. B. Navi , et al., “Tumor Genomic Profile Is Associated With Arterial Thromboembolism Risk in Patients With Solid Cancer,” JACC CardioOncology 5, no. 2 (2023): 246–255, 10.1016/j.jaccao.2023.01.009. 37144118 PMC10152200

[19] A. Leader , O. Icht , E. Batat , et al., “Incidence and Risk Factors of Arterial Thromboembolism in Non‐Small Cell Lung Cancer,” Blood 134 (2019): 3654, 10.1182/blood-2019-128822.

[20] S. Schneeweiss , J. A. Rassen , J. S. Brown , et al., “Graphical Depiction of Longitudinal Study Designs in Health Care Databases,” Annals of Internal Medicine 170, no. 6 (2019): 398–406, 10.7326/M18-3079. 30856654

[21] K. Nagai , T. Tanaka , N. Kodaira , S. Kimura , Y. Takahashi , and T. Nakayama , “Data Resource Profile: JMDC Claims Database Sourced From Health Insurance Societies,” Journal of General and Family Medicine 22, no. 3 (2021): 118–127, 10.1002/jgf2.422. 33977008 PMC8090843

[22] A. Okada and H. Yasunaga , “Overview of the DeSC Database and Utilization for Clinical Epidemiology and Pharmacoepidemiology Research,” Japanese Journal of Pharmacoepidemiology 27 (2022): 11–18.

[23] N. Breslow , “Covariance Analysis of Censored Survival Data,” Biometrics 30, no. 1 (1974): 89–99. 4813387

[24] J. Herrmann , “Vascular Toxic Effects of Cancer Therapies,” Nature Reviews. Cardiology 17, no. 8 (2020): 503–522, 10.1038/s41569-020-0347-2. 32218531 PMC8782612

[25] E. H. Yang , K. Marmagkiolis , D. V. Balanescu , et al., “Radiation‐Induced Vascular Disease—A State‐Of‐The‐Art Review,” Frontiers in Cardiovascular Medicine 8 (2021): 652761, 10.3389/fcvm.2021.652761. 33860001 PMC8042773

[26] Q. Wang , Z. H. Gümüş , C. Colarossi , et al., “SCLC: Epidemiology, Risk Factors, Genetic Susceptibility, Molecular Pathology, Screening, and Early Detection,” Journal of Thoracic Oncology 18, no. 1 (2023): 31–46, 10.1016/j.jtho.2022.10.002. 36243387 PMC10797993

