# Supplementary material for: Arterial Thromboembolism Incidence in Japanese Patients With Lung Cancer
Source: Cancer Med. 2025 Nov 20;14(22):e71356. doi: 10.1002/cam4.71356 (PMC12631742; doi:10.1002/cam4.71356)
Supplement: Supplementary file 1 — Data S1: cam471356‐sup‐0001‐supinfo.docx. [file CAM4-14-e71356-s001.docx]

**Supplementary Material**

**Arterial Thromboembolism Incidence in Japanese Patients with Lung Cancer**

**Table of Contents**

| **Title** | **Description** | **Page(s)** |
| --- | --- | --- |
| **List of lung cancer** | **Table S1** | **2** |
| **Algorithms for treatment pattern** | **Table S2** | **4** |
| **Algorithms for systemic therapy subtype** | **Table S3** | **5** |
| **Definition of covariates for Cox regression model** | **Table S4** | **6** |
| **Cumulative incidence rate (95% CI*) of ATE by years after lung cancer diagnosis** | **Table S5** | **7** |
| **Treatment pattern by histological subtype and NSCLC subtype** | **Table S6** | **9** |
| **Sensitivity analysis** | **Table S7** | **10** |
| **Flow diagram of patient selection** | **Figure S1** | **13** |

**Table S1. List of lung cancer**

| **Histological subtype** | **NSCLC subtype** | **Disease code** | **Disease name** |
| --- | --- | --- | --- |
| Non-small cell cancer lung | Squamous cell carcinoma | 8847679 | hilar squamous cell carcinoma |
| Non-small cell cancer lung | Squamous cell carcinoma | 8847636 | superior lobe squamous cell carcinoma of lung |
| Non-small cell cancer lung | Squamous cell carcinoma | 8847663 | middle lobe squamous cell lung cancer |
| Non-small cell cancer lung | Squamous cell carcinoma | 8847597 | inferior lobe squamous cell carcinoma of lung |
| Non-small cell cancer lung | Squamous cell carcinoma | 8838898 | squamous cell carcinoma of lung |
| Non-small cell cancer lung | Large cell lung cancer | 8847677 | hilar large cell cancer |
| Non-small cell cancer lung | Large cell lung cancer | 8847635 | superior lobe large cell lung cancer |
| Non-small cell cancer lung | Large cell lung cancer | 8847662 | middle lobe large cell lung cancer |
| Non-small cell cancer lung | Large cell lung cancer | 8847596 | inferior lobe large cell lung cancer |
| Non-small cell cancer lung | Large cell lung cancer | 8838852 | large cell lung cancer |
| Non-small cell cancer lung | Large cell lung cancer | 8842057 | pulmonary large cell neuroendocrine carcinoma |
| Non-small cell cancer lung | Adenocarcinoma | 8847676 | hilar adenocarcinoma |
| Non-small cell cancer lung | Adenocarcinoma | 8847634 | superior lobe lung adenocarcinoma |
| Non-small cell cancer lung | Adenocarcinoma | 8847661 | middle lobe lung adenocarcinoma |
| Non-small cell cancer lung | Adenocarcinoma | 8847595 | inferior lobe lung adenocarcinoma |
| Non-small cell cancer lung | Adenocarcinoma | 8833932 | bronchioloalveolar cell carcinoma |
| Non-small cell cancer lung | Adenocarcinoma | 8838901 | alveolar cell carcinoma |
| Non-small cell cancer lung | Adenocarcinoma | 8838844 | lung adenocarcinoma |
| Non-small cell cancer lung | Other | 8847678 | hilar non-small cell cancer |
| Non-small cell cancer lung | Other | 8847637 | superior lobe non-small cell lung cancer |
| Non-small cell cancer lung | Other | 8847664 | middle lobe non-small cell lung cancer |
| Non-small cell cancer lung | Other | 8847598 | inferior lobe non-small cell lung cancer |
| Non-small cell cancer lung | Other | 8847272 | ALK fusion gene positive non-small cell lung cancer |
| Non-small cell cancer lung | Other | 8849238 | ROS1 fusion gene positive non-small cell lung cancer |
| Non-small cell cancer lung | Other | 8842053 | non-small cell lung cancer |
| Non-small cell cancer lung | Other | 8847732 | EGFR mutation positive advanced non-small cell lung cancer |
| Small cell lung cancer |  | 8847675 | hilar small cell cancer |
| Small cell lung cancer |  | 8847633 | superior lobe small cell lung cancer |
| Small cell lung cancer |  | 8847660 | middle lobe small cell lung cancer |
| Small cell lung cancer |  | 8847594 | inferior lobe small cell lung cancer |
| Small cell lung cancer |  | 8842185 | small cell lung cancer |
| Unknown |  | 8842835 | hilar lung cancer |
| Unknown |  | 8834569 | malignant main bronchial tumor |
| Unknown |  | 8835493 | lung cancer of superior lobe |
| Unknown |  | 8845183 | Pancoast's syndrome |
| Unknown |  | 8837666 | lung cancer of middle lobe |
| Unknown |  | 8831458 | lung cancer of inferior lobe |
| Unknown |  | 1629006 | lung cancer |
| Unknown |  | 8847242 | obstructive pneumonia due to lung cancer |
| Unknown |  | 8849759 | pediatric pleuropulmonary blastoma |
| Unknown |  | 8850564 | lung malignant melanoma |
| Unknown |  | 1629003 | primary lung cancer |
| Unknown |  | 1629009 | pulmonary sarcoma |
| Unknown |  | 8832157 | bronchial carcinoma |
| Unknown |  | 8842831 | lung carcinosarcoma |
| Unknown |  | 8842832 | adenosquamous carcinoma of the lung |
| Unknown |  | 8842834 | pulmonary mucoepidermoid carcinoma |
| Unknown |  | 8846553 | bronchial carcinoid |
| Unknown |  | 8848775 | pulmonary choriocarcinoma |
| Unknown |  | 8849788 | adult type pulmonary blastoma |
| Unknown |  | 8838804 | pulmonary carcinoid |
| Unknown |  | 8838904 | pulmonary undifferentiated carcinoma |
| Unknown |  | 8842833 | pulmonary adenoid cystic cancer |

**Table S2. Algorithms for treatment pattern**

| **Base treatment** | **Condition of base treatment** | **Start date** | **End date** | **Combined treatment** | **Treatment pattern** |
| --- | --- | --- | --- | --- | --- |
| 1) Radiation therapy | Patient had a history of radiotherapy within 6 months of the start date | Index date | Adopted the longest of the following dates: 1) If the last treatment date was after 365 days from the start date, The end date was the start date + 364 days 2) Last treatment date + 89 days 3) Start date + 179 days | Surgery was performed during the base treatment period. | Others |
|  |  |  |  | Systemic treatment was performed during the base treatment period without the above condition. | Radiation therapy + Systemic therapy |
|  |  |  |  | Other than above | Radiation therapy only |
| 2) Surgery | Patient did not meet base treatment 1) and had a first surgery within 6 months of the start date | Index date | Adopted the longest of the following dates: 1) Date of first surgery + 89 days 2) If the date of 1) was less than 180 days, the end date was the start date + 179 days | Radiation therapy was performed during the base treatment period. | Others |
|  |  |  |  | Systemic treatment was performed during the base treatment period without the above condition. | Surgery + Systemic therapy |
|  |  |  |  | Other than above | Surgery only |
| 3) Systemic treatment | Patient did not meet base treatment 1) and 2) and had a history of systemic treatment within 6 months of the start date | NA | NA | Condition of base treatment was met. | Systemic therapy only |
| 4) Other | Patient did not meet base treatment 1), 2), and 3), and had a history of other treatment within 6 months of the start date | NA | NA | Condition of base treatment was met. | Others |

**Table S3. Algorithms for systemic therapy subtype**

| **Systemic therapy subtype** | **Target population** | **Definition** |
| --- | --- | --- |
| 1) Other therapy | Patient with systemic therapy only | Patient had a history of other systemic therapy only within 6 months of index date |
| 2) Chemotherapy |  | patient did not meet 1), and had a history of chemotherapy only within 6 months of index date |
| 3) Molecularly targeted therapy |  | Patient did not meet 1), and had a history of molecularly targeted therapy only within 6 months of index date |
| 4) Immune checkpoint inhibitor |  | Patient did not meet 1), and had a history of immune checkpoint inhibitor only within 6 months of index date |
| 5) Multiple therapy |  | Other than above |

**Table S4. Definition of covariates for Cox regression model**

| **Covariate** | **Histological subtype** | **Treatment Pattern/Predictive factor** |
| --- | --- | --- |
| Age | ○ | ○ |
| Sex (**male**, female) | ○ | ○ |
| Histological subtypes (**NSCLC**, SCLC, unknown) |  | ○ |
| Heart failure (Yes, **No**) | ○ | ○ |
| Hypertension (Yes, **No**) | ○ | ○ |
| Atrial fibrillation/flutter (Yes, **No**) | ○ | ○ |
| Coronary artery disease (Yes, **No**) | ○ | ○ |
| Venous thromboembolism (Yes, **No**) | ○ | ○ |
| Chronic kidney disease (Yes, **No**) | ○ | ○ |
| Diabetes mellitus (Yes, **No**) | ○ | ○ |
| Dyslipidemia (Yes, **No**) | ○ | ○ |
| Chronic obstructive pulmonary disease (Yes, **No**) | ○ | ○ |
| Dementia (Yes, **No**) | ○ | ○ |
| History of ATE (Yes, **No**) | ○ | ○ |

Bold font: Reference

**Table S5. Cumulative incidence rate (95% CI ^†^) of ATE by years after lung cancer diagnosis**

|  | **3-month** | **6-month** | **1-year** | **2-year** | **3-year** | **4-year** | **5-year** |
| --- | --- | --- | --- | --- | --- | --- | --- |
| **EHI** |  |  |  |  |  |  |  |
| All patients | 1.1 [0.85–1.43] | 1.5 [1.21–1.91] | 1.7 [1.39– 2.14] | 2.4 [1.93–2.91] | 2.8 [2.25–3.44] | 3.0 [2.41–3.75] | 3.5 [2.66–4.53] |
| Histological subtype |  |  |  |  |  |  |  |
| NSCLC | 1.0 [0.70–1.54] | 1.3 [0.87–1.82] | 1.5 [1.03–2.08] | 1.7 [1.16–2.34] | 2.2 [1.47–3.16] | 2.2 [1.47–3.16] | 3.4 [1.94–5.89] |
| SCLC | 2.5 [1.22–5.01] | 3.7 [1.93–7.20] | 4.9 [2.48–9.54] | 7.2 [3.32–15.29] | 7.2 [3.32–15.29] | 7.2 [3.32–15.29] | - |
| Unknown | 1.0 [0.68–1.47] | 1.5 [1.10–2.09] | 1.7 [1.23–2.28] | 2.6 [2.00–3.45] | 3.0 [2.26–3.91] | 3.3 [2.50–4.46] | 3.3 [2.50–4.46] |
| NSCLC subtype |  |  |  |  |  |  |  |
| Adenocarcinoma | 0.8 [0.48–1.49] | 0.9 [0.54–1.63] | 0.9 [0.54–1.63] | 1.1 [0.62–1.81] | 1.3 [0.74–2.30] | 1.3 [0.74–2.30] | 3.1 [1.34–7.02] |
| Squamous cell carcinoma | 1.2 [0.38–3.67] | 1.7 [0.64–4.54] | 2.3 [0.95–5.55] | 2.3 [0.95–5.55] | 6.9 [2.55–17.80] | 6.9 [2.55–17.80] | 6.9 [2.55–17.80] |
| Large cell carcinoma | 0.0 [0.00–0.00] | 0.0 [0.00–0.00] | 0.0 [0.00–0.00] | 0.0 [0.00–0.00] | 0.0 [0.00–0.00] | - | - |
| Others | 1.4 [0.74–2.58] | 1.8 [1.00–3.14] | 2.3 [1.31–3.84] | 2.7 [1.55–4.60] | 2.7 [1.55–4.60] | 2.7 [1.55–4.60] | 2.7 [1.55–4.60] |
| Treatment Pattern |  |  |  |  |  |  |  |
| Surgery only | 0.5 [0.27–0.92] | 0.7 [0.42–1.20] | 0.9 [0.55–1.42] | 1.3 [0.83–1.93] | 1.5 [0.97–2.22] | 1.7 [1.09–2.61] | 2.1 [1.24–3.41] |
| Surgery + Systemic therapy | 0.3 [0.08–0.77] | 0.7 [0.35–1.40] | 0.8 [0.42–1.56] | 0.9 [0.50–1.75] | 1.8 [0.95–3.32] | 1.8 [0.95–3.32] | 1.8 [0.95–3.32] |
| Radiation therapy only | 2.7 [0.83–8.68] | 2.7 [0.83–8.68] | 2.7 [0.83–8.68] | 5.9 [1.80–18.10] | 5.9 [1.80–18.10] | 5.9 [1.80–18.10] | 5.9 [1.80–18.10] |
| Radiation therapy + Systemic therapy | 2.5 [1.60–3.91] | 2.7 [1.76–4.26] | 2.7 [1.76–4.26] | 3.2 [2.01–5.21] | 3.2 [2.01–5.21] | 6.2 [2.36–15.60] | 6.2 [2.36–15.60] |
| Systemic therapy only | 2.0 [1.26–3.14] | 3.1 [2.06–4.64] | 3.9 [2.61–5.76] | 7.6 [5.20–11.09] | 8.3 [5.66–12.14] | 8.3 [5.66–12.14] | 12.7 [6.21–24.96] |
| Others | 1.9 [0.47–7.39] | 3.0 [0.97–9.04] | 3.0 [0.97–9.04] | 3.0 [0.97–9.04] | 3.0 [0.97–9.04] | 3.0 [0.97–9.04] | 3.0 [0.97–9.04] |
| **NHI** |  |  |  |  |  |  |  |
| All patients | 3.2 [2.88–3.64] | 3.8 [3.42–4.27] | 4.5 [4.08–5.04] | 5.4 [4.81–5.96] | 6.4 [5.68–7.16] | 7.5 [6.56–8.53] | 8.0 [6.85–9.32] |
| Histological subtype |  |  |  |  |  |  |  |
| NSCLC | 3.7 [3.11–4.31] | 4.1 [3.49–4.80] | 4.8 [4.08–5.56] | 5.5 [4.68–6.40] | 6.5 [5.46–7.77] | 8.1 [6.48–10.03] | 9.7 [7.14–13.06] |
| SCLC | 2.9 [1.95–4.39] | 3.7 [2.48–5.44] | 6.0 [4.05–8.82] | 9.2 [5.67–14.58] | 9.2 [5.67–14.58] | 13.7 [6.68–26.91] | 13.7 [6.68–26.91] |
| Unknown | 2.9 [2.38–3.44] | 3.6 [2.99–4.20] | 4.1 [3.52–4.86] | 4.9 [4.19–5.76] | 6.0 [5.04–7.03] | 6.7 [5.61–7.95] | 6.7 [5.61–7.95] |
| NSCLC subtype |  |  |  |  |  |  |  |
| Adenocarcinoma | 3.2 [2.52–4.08] | 3.7 [2.91–4.63] | 4.1 [3.27–5.14] | 5.0 [3.96–6.26] | 6.2 [4.84–7.98] | 7.7 [5.71–10.21] | 8.4 [6.11–11.46] |
| Squamous cell carcinoma | 4.6 [3.30–6.26] | 5.1 [3.74–6.93] | 6.0 [4.46–8.11] | 6.5 [4.76–8.87] | 8.3 [5.61–12.19] | 12.5 [7.39–20.64] | 19.8 [9.04–40.06] |
| Large cell carcinoma | 0.0 [0.00–0.00] | 0.0 [0.00–0.01] | 0.0 [0.00–0.02] | 0.0 [0.00–0.03] | 0.0 [0.00–0.04] | - | - |
| Others | 3.9 [2.85–5.29] | 4.2 [3.09–5.67] | 5.2 [3.86–6.98] | 5.7 [4.24–7.64] | 5.7 [4.24–7.64] | 5.7 [4.24–7.64] | 5.7 [4.24–7.64] |
| Treatment Pattern |  |  |  |  |  |  |  |
| Surgery only | 1.9 [1.41–2.51] | 2.3 [1.79–3.01] | 2.9 [2.25–3.61] | 3.3 [2.66–4.18] | 3.9 [3.13–4.92] | 4.9 [3.87–6.28] | 5.8 [4.31–7.85] |
| Surgery + Systemic therapy | 2.2 [1.52–3.16] | 2.8 [2.02–3.88] | 3.2 [2.33–4.34] | 4.1 [3.09–5.54] | 5.0 [3.71–6.67] | 5.0 [3.71–6.67] | 5.0 [3.71–6.67] |
| Radiation therapy only | 6.2 [4.25–9.06] | 6.2 [4.25–9.06] | 7.7 [5.13–11.40] | 12.2 [7.51–19.40] | 12.2 [7.51–19.40] | 12.2 [7.51–19.40] | 12.2 [7.51–19.40] |
| Radiation therapy + Systemic therapy | 3.6 [2.82–4.67] | 4.0 [3.12–5.15] | 5.1 [3.93–6.60] | 5.9 [4.50–7.72] | 9.0 [5.90–13.71] | 17.8 [11.13–27.75] | 17.8 [11.13–27.75] |
| Systemic therapy only | 4.2 [3.44–5.02] | 5.0 [4.19–6.04] | 6.3 [5.25–7.59] | 7.5 [6.12–9.24] | 10.4 [7.86–13.78] | 10.4 [7.86–13.78] | 10.4 [7.86–13.78] |
| Others | 2.0 [0.62–6.06] | 3.9 [1.61–9.43] | 3.9 [1.61–9.43] | 3.9 [1.61–9.43] | 3.9 [1.61–9.43] | 3.9 [1.61–9.43] | 3.9 [1.61–9.43] |
| **LSEHS** |  |  |  |  |  |  |  |
| All patients | 4.6 [4.25–5.06] | 5.8 [5.33–6.25] | 7.2 [6.68–7.75] | 9.2 [8.55–9.88] | 11.3 [10.41–12.17] | 12.8 [11.65–14.00] | 15.9 [13.81–18.23] |
| Histological subtype |  |  |  |  |  |  |  |
| NSCLC | 4.8 [4.22–5.50] | 5.8 [5.16–6.59] | 7.2 [6.46–8.11] | 9.7 [8.72–10.88] | 12.2 [10.79–13.77] | 12.9 [11.33–14.75] | 17.0 [12.76–22.39] |
| SCLC | 6.7 [5.07–8.75] | 7.6 [5.80–9.85] | 9.8 [7.44–12.74] | 11.3 [8.37–15.17] | 11.3 [8.37–15.17] | 11.3 [8.37–15.17] | 11.3 [8.37–15.17] |
| Unknown | 4.2 [3.69–4.78] | 5.5 [4.85–6.12] | 6.9 [6.15–7.62] | 8.5 [7.67–9.41] | 10.4 [9.32–11.57] | 12.3 [10.85–14.00] | 15.2 [12.79–18.07] |
| NSCLC subtype |  |  |  |  |  |  |  |
| Adenocarcinoma | 4.3 [3.50–5.22] | 5.6 [4.64–6.65] | 7.3 [6.21–8.59] | 9.6 [8.17–11.17] | 11.7 [9.92–13.86] | 11.7 [9.92–13.86] | 16.6 [11.20–24.21] |
| Squamous cell carcinoma | 6.5 [5.13–8.22] | 7.3 [5.80–9.11] | 8.6 [6.93–10.67] | 12.3 [9.96–15.19] | 16.8 [13.04–21.45] | 18.9 [14.45–24.52] | 21.9 [15.42–30.58] |
| Large cell carcinoma | 9.8 [2.49–34.39] | 9.8 [2.49–34.39] | 9.8 [2.49–34.39] | 9.8 [2.49–34.39] | 9.8 [2.49–34.39] | - | - |
| Others | 4.4 [3.34–5.67] | 5.1 [3.98–6.56] | 6.0 [4.71–7.55] | 8.1 [6.37–10.25] | 9.5 [7.42–12.18] | 11.8 [7.71–17.80] | 11.8 [7.71–17.80] |
| Treatment Pattern |  |  |  |  |  |  |  |
| Surgery only | 3.7 [3.10–4.33] | 4.6 [3.94–5.32] | 5.6 [4.90–6.43] | 7.3 [6.46–8.31] | 9.2 [8.05–10.45] | 10.8 [9.31–12.52] | 13.5 [11.06–16.37] |
| Surgery + Systemic therapy | 3.0 [2.01–4.37] | 4.0 [2.86–5.59] | 4.4 [3.21–6.08] | 5.8 [4.32–7.88] | 8.1 [6.04–10.89] | 9.4 [6.83–12.74] | 9.4 [6.83–12.74] |
| Radiation therapy only | 4.4 [3.53–5.58] | 5.8 [4.71–7.13] | 8.3 [6.93–9.96] | 11.1 [9.34–13.26] | 13.8 [11.35–16.66] | 15.2 [12.22–18.70] | 24.4 [15.45–37.37] |
| Radiation therapy + Systemic therapy | 5.2 [4.00–6.66] | 5.8 [4.54–7.50] | 8.4 [6.57–10.77] | 10.5 [8.17–13.46] | 11.3 [8.62–14.67] | 11.3 [8.62–14.67] | 11.3 [8.62–14.67] |
| Systemic therapy only | 5.9 [5.12–6.77] | 7.4 [6.47–8.43] | 9.1 [7.98–10.30] | 11.5 [10.06–13.23] | 14.2 [12.09–16.74] | 16.3 [12.90–20.38] | 23.2 [14.70–35.40] |
| Others | 2.8 [0.90–8.47] | 6.0 [2.44–14.33] | 8.2 [3.58–18.35] | 11.1 [5.05–23.45] | 11.1 [5.05–23.45] | 11.1 [5.05–23.45] | - |

ATE, arterial thromboembolism; CI, confidence interval; NSCLC, non-small cell lung cancer; SCLC, small cell lung cancer.

^†^ Nelson-Aalen's confidence interval

**Table S6. Treatment pattern by histological subtype and NSCLC subtype**

| **Treatment pattern** | **NSCLC** | | | | | **SCLC** | **Unknown** |
| --- | --- | --- | --- | --- | --- | --- | --- |
|  | **All** | **Adenocarcinoma** | **Squamous cell carcinoma** | **Large cell carcinoma** | **Other** |  |  |
| **EHI** | 2,954 | 1,722 | 344 | 28 | 860 | 399 | 2,987 |
| Surgery only | 700 (23.7) | 477 (27.7) | 46 (13.4) | 1 (3.6) | 176 (20.5) | 6 (1.5) | 1,400 (46.9) |
| Surgery + Systemic therapy | 607 (20.5) | 364 (21.1) | 65 (18.9) | 7 (25.0) | 171 (19.9) | 17 (4.3) | 635 (21.3) |
| Radiation therapy only | 51 (1.7) | 22 (1.3) | 11 (3.2) | 0 (0.0) | 18 (2.1) | 4 (1.0) | 92 (3.1) |
| Radiation therapy + Systemic therapy | 681 (23.1) | 335 (19.5) | 113 (32.8) | 12 (42.9) | 221 (25.7) | 196 (49.1) | 324 (10.8) |
| Systemic therapy only | 843 (28.5) | 497 (28.9) | 94 (27.3) | 6 (21.4) | 246 (28.6) | 172 (43.1) | 479 (16.0) |
| Others | 72 (2.4) | 27 (1.6) | 15 (4.4) | 2 (7.1) | 28 (3.3) | 4 (1.0) | 57 (1.9) |
| **NHI** | 4,969 | 2,588 | 1031 | 50 | 1300 | 1019 | 4,869 |
| Surgery only | 811 (16.3) | 498 (19.2) | 119 (11.5) | 5 (10.0) | 189 (14.5) | 10 (1.0) | 1,794 (36.8) |
| Surgery + Systemic therapy | 625 (12.6) | 347 (13.4) | 100 (9.7) | 5 (10.0) | 173 (13.3) | 36 (3.5) | 710 (14.6) |
| Radiation therapy only | 217 (4.4) | 104 (4.0) | 72 (7.0) | 1 (2.0) | 40 (3.1) | 20 (2.0) | 321 (6.6) |
| Radiation therapy + Systemic therapy | 1375 (27.7) | 604 (23.3) | 366 (35.5) | 16 (32.0) | 389 (29.9) | 399 (39.2) | 808 (16.6) |
| Systemic therapy only | 1845 (37.1) | 998 (38.6) | 347 (33.7) | 23 (46.0) | 477 (36.7) | 551 (54.1) | 1,130 (23.2) |
| Others | 96 (1.9) | 37 (1.4) | 27 (2.6) | 0 (0.0) | 32 (2.5) | 3 (0.3) | 106 (2.2) |
| **LSEHS** | 5,701 | 2,824 | 1,214 | 24 | 1,639 | 1,026 | 6,312 |
| Surgery only | 1,380 (24.2) | 756 (26.8) | 252 (20.8) | 5 (20.8) | 367 (22.4) | 16 (1.6) | 2,429 (38.5) |
| Surgery + Systemic therapy | 360 (6.3) | 213 (7.5) | 49 (4.0) | 2 (8.3) | 96 (5.9) | 35 (3.4) | 515 (8.2) |
| Radiation therapy only | 777 (13.6) | 338 (12.0) | 227 (18.7) | 2 (8.3) | 210 (12.8) | 63 (6.1) | 1,135 (18.0) |
| Radiation therapy + Systemic therapy | 969 (17.0) | 413 (14.6) | 245 (20.2) | 7 (29.2) | 304 (18.5) | 287 (28.0) | 539 (8.5) |
| Systemic therapy only | 2,158 (37.9) | 1,088 (38.5) | 424 (34.9) | 8 (33.3) | 638 (38.9) | 621 (60.5) | 1,635 (25.9) |
| Others | 57 (1.0) | 16 (0.6) | 17 (1.4) | 0 (0.0) | 24 (1.5) | 4 (0.4) | 59 (0.9) |

NSCLC, non-small cell lung cancer; SCLC, small cell lung cancer.

**Table S7. Sensitivity analysis**

|  | **Including patient with a history of ATE** | | | | **Excluding patient with a history of ATE** | | | |
| --- | --- | --- | --- | --- | --- | --- | --- | --- |
|  | **Patients, n** | **Event, n (%)** | **Adjusted hazard ratio^†^** | **95% CI ^‡^** | **Patients, n** | **Event, n (%)** | **Adjusted hazard ratio^†^** | **95% CI ^‡^** |
| **EHI** |  |  |  |  |  |  |  |  |
| Histological subtype |  |  |  |  |  |  |  |  |
| NSCLC | 2,954 | 39 (1.3) | Reference |  | 2,874 | 29 (1.0) | Reference |  |
| SCLC | 399 | 12 (3.0) | 2.84 | [1.46–5.54] | 385 | 7 (1.8) | 2.36 | [1.02–5.48] |
| Unknown | 2,987 | 59 (2.0) | 1.18 | [0.78–1.77] | 2,888 | 39 (1.4) | 1.06 | [0.65–1.72] |
| NSCLC subtype |  |  |  |  |  |  |  |  |
| Adenocarcinoma | 1,722 | 17 (1.0) | Reference |  | 1,679 | 13 (0.8) | Reference |  |
| Squamous cell carcinoma | 344 | 7 (2.0) | 1.68 | [0.68–4.19] | 336 | 6 (1.8) | 2.00 | [0.74–5.43] |
| Large cell carcinoma | 28 | 0 (0.0) | - |  | 27 | 0 (0.0) | - |  |
| Others | 860 | 15 (1.7) | 1.64 | [0.80–3.33] | 832 | 10 (1.2) | 1.65 | [0.72–3.81] |
| Treatment Pattern |  |  |  |  |  |  |  |  |
| Surgery only | 2,106 | 26 (1.2) | Reference |  | 2,047 | 15 (0.7) | Reference |  |
| Surgery + Systemic therapy | 1,259 | 13 (1.0) | 0.99 | [0.50–1.93] | 1,234 | 10 (0.8) | 1.26 | [0.56–2.81] |
| RT only | 147 | 4 (2.7) | 1.94 | [0.60–6.22] | 137 | 3 (2.2) | 5.13 | [1.30–20.25] |
| RT + Systemic therapy | 1,201 | 24 (2.0) | 3.59 | [1.95–6.64] | 1,157 | 17 (1.5) | 5.74 | [2.70–12.18] |
| Systemic therapy only | 1,494 | 40 (2.7) | 5.34 | [3.11–9.17] | 1,442 | 27 (1.9) | 6.93 | [3.52–13.66] |
| Others | 133 | 3 (2.3) | 3.32 | [0.99–11.13] | 130 | 3 (2.3) | 4.62 | [1.32–16.12] |
| Systemic therapy subtype |  |  |  |  |  |  |  |  |
| Chemotherapy | 570 | 40 (2.7) | Reference |  | 546 | 10 (1.8) | Reference |  |
| Molecularly targeted therapy | 313 | 16 (2.8) | 1.15 | [0.40–3.30] | 304 | 4 (1.3) | 1.06 | [0.31–3.65] |
| Immune checkpoint inhibitor | 63 | 6 (1.9) | 1.42 | [0.31–6.52] | 60 | 2 (3.3) | 1.52 | [0.31–7.44] |
| Multiple therapy | 497 | 2 (3.2) | 1.49 | [0.69–3.22] | 483 | 10 (2.1) | 1.57 | [0.64–3.85] |
| Other therapy | 51 | 14 (2.8) | 1.72 | [0.32–9.41] | 49 | 1 (2.0) | 2.39 | [0.27–21.06] |
| **NHI** |  |  |  |  |  |  |  |  |
| Histological subtype |  |  |  |  |  |  |  |  |
| NSCLC | 4,969 | 193 (3.9) | Reference |  | 4,661 | 133 (2.9) | Reference |  |
| SCLC | 1,019 | 37 (3.6) | 0.93 | [0.65–1.32] | 933 | 16 (1.7) | 0.65 | [0.39–1.10] |
| Unknown | 4,869 | 179 (3.7) | 0.77 | [0.63–0.94] | 4,524 | 104 (2.3) | 0.71 | [0.55–0.92] |
| NSCLC subtype |  |  |  |  |  |  |  |  |
| Adenocarcinoma | 2,588 | 94 (3.6) | Reference |  | 2,440 | 63 (2.6) | Reference |  |
| Squamous cell carcinoma | 1,031 | 50 (4.8) | 1.06 | [0.75–1.52] | 956 | 36 (3.8) | 1.26 | [0.82–1.92] |
| Large cell carcinoma | 50 | 0 (0.0) | - |  | 47 | 0 (0.0) | - |  |
| Others | 1,300 | 49 (3.8) | 1.00 | [0.70–1.41] | 1,218 | 34 (2.8) | 1.09 | [0.72–1.66] |
| Treatment Pattern |  |  |  |  |  |  |  |  |
| Surgery only | 2,615 | 89 (3.4) | Reference |  | 2,439 | 54 (2.2) | Reference |  |
| Surgery + Systemic therapy | 1,371 | 50 (3.6) | 1.18 | [0.83–1.67] | 1,286 | 33 (2.6) | 1.16 | [0.75–1.79] |
| RT only | 558 | 33 (5.9) | 2.91 | [1.92–4.39] | 510 | 27 (5.3) | 4.53 | [2.80–7.33] |
| RT + Systemic therapy | 2,582 | 85 (3.3) | 2.06 | [1.49–2.84] | 2,403 | 52 (2.2) | 2.04 | [1.35–3.08] |
| Systemic therapy only | 3,526 | 147 (4.2) | 2.46 | [1.85–3.29] | 3,285 | 83 (2.5) | 2.12 | [1.46–3.07] |
| Others | 205 | 5 (2.4) | 1.25 | [0.50–3.08] | 195 | 4 (2.1) | 1.30 | [0.47–3.61] |
| Systemic therapy subtype |  |  |  |  |  |  |  |  |
| Chemotherapy | 1,401 | 55 (3.9) | Reference |  | 1,293 | 24 (1.9) | Reference |  |
| Molecularly targeted therapy | 572 | 16 (2.8) | 0.85 | [0.47–1.56] | 546 | 10 (1.8) | 1.03 | [0.47–2.28] |
| Immune checkpoint inhibitor | 197 | 11 (5.6) | 1.38 | [0.70–2.71] | 184 | 5 (2.7) | 1.09 | [0.41–2.95] |
| Multiple therapy | 1,198 | 55 (4.6) | 1.10 | [0.74–1.64] | 1,122 | 37 (3.3) | 1.53 | [0.89–2.61] |
| Other therapy | 158 | 10 (6.3) | 2.16 | [1.06–4.42] | 140 | 7 (5.0) | 4.53 | [1.86–11.03] |
| **LSEHS** |  |  |  |  |  |  |  |  |
| Histological subtype |  |  |  |  |  |  |  |  |
| NSCLC | 5,701 | 369 (6.5) | Reference |  | 4,942 | 218 (4.4) | Reference |  |
| SCLC | 1,026 | 63 (6.1) | 1.11 | [0.85–1.46] | 857 | 24 (2.8) | 0.83 | [0.55–1.27] |
| Unknown | 6,312 | 416 (6.6) | 0.84 | [0.73–0.97] | 5,357 | 205 (3.8) | 0.74 | [0.61–0.90] |
| NSCLC subtype |  |  |  |  |  |  |  |  |
| Adenocarcinoma | 2,824 | 179 (6.3) | Reference |  | 2,495 | 110 (4.4) | Reference |  |
| Squamous cell carcinoma | 1,214 | 105 (8.6) | 1.18 | [0.92–1.53] | 1,015 | 56 (5.5) | 1.19 | [0.84–1.67] |
| Large cell carcinoma | 24 | 2 (8.3) | 1.24 | [0.31–5.04] | 20 | 1 (5.0) | 0.95 | [0.13–6.88] |
| Others | 1,639 | 83 (5.1) | 0.79 | [0.61–1.03] | 1,412 | 51 (3.6) | 0.85 | [0.61–1.19] |
| Treatment Pattern |  |  |  |  |  |  |  |  |
| Surgery only | 3,825 | 273 (7.1) | Reference |  | 3,274 | 138 (4.2) | Reference |  |
| Surgery + Systemic therapy | 910 | 52 (5.7) | 0.93 | [0.69–1.25] | 809 | 30 (3.7) | 0.91 | [0.62–1.36] |
| RT only | 1,975 | 146 (7.4) | 1.24 | [1.00–1.52] | 1,585 | 74 (4.7) | 1.47 | [1.09–1.98] |
| RT + Systemic therapy | 1,795 | 84 (4.7) | 1.46 | [1.13–1.89] | 1,560 | 48 (3.1) | 1.65 | [1.17–2.33] |
| Systemic therapy only | 4,414 | 286 (6.5) | 1.75 | [1.47–2.09] | 3,818 | 153 (4.0) | 1.90 | [1.49–2.42] |
| Others | 120 | 7 (5.8) | 1.67 | [0.79–3.55] | 110 | 4 (3.6) | 1.43 | [0.53–3.87] |
| Systemic therapy subtype |  |  |  |  |  |  |  |  |
| Chemotherapy | 1,642 | 119 (7.2) | Reference |  | 1,406 | 68 (4.8) | Reference |  |
| Molecularly targeted therapy | 936 | 42 (4.5) | 0.67 | [0.45–1.00] | 831 | 25 (3.0) | 0.51 | [0.30–0.87] |
| Immune checkpoint inhibitor | 372 | 33 (8.9) | 0.89 | [0.59–1.33] | 318 | 15 (4.7) | 0.67 | [0.38–1.20] |
| Multiple therapy | 774 | 52 (6.7) | 0.93 | [0.66–1.30] | 685 | 28 (4.1) | 0.79 | [0.50–1.25] |
| Other therapy | 690 | 40 (5.8) | 0.72 | [0.47–1.09] | 578 | 17 (2.9) | 0.59 | [0.32–1.09] |

CI, confidence interval; NSCLC, non-small cell lung cancer; SCLC, small cell lung cancer; RT, Radiation therapy. Other abbreviations are shown in Table 1.

† Adjusted for sex, age at cancer diagnosis, histological subtype, brain metastasis, treatment pattern, comorbidities (heart failure, hypertension, atrial fibrillation/flutter, coronary artery disease, venous thromboembolism, chronic kidney disease, diabetes mellitus, dyslipidemia, chronic obstructive pulmonary disease, and dementia), and history of ATE.

‡ Wald's confidence interval

**Figure S1. Flow diagram of patient selection**

| All Patients who were diagnosed lung cancer (ICD-10 codes: C340, C341, C342, C343, C348, C349) from April 1, 2014, to March 31, 2021 | EHI: n=23,944 (100.0%)  NHI: n=42,438 (100.0%)  LSEHS: n=106,915 (100.0%) |  |  | |
| --- | --- | --- | --- | --- |
|  |  |  |  | |
| Patients meeting all inclusion criteria ^†^ | EHI: n=8,259 (34.5%)  NHI: n=14,048 (33.1%)  LSEHS: n=18,936 (17.7%) |  |  | |
| Patients who received at least one treatment for lung cancer within six months from and on the index date ^‡^ | EHI: n=10,146  NHI: n=19,362  LSEHS: n=27,556 |  |  | |
| Patients who were >= 20 years old at the index date ^‡^ | EHI: n=23,871  NHI: n=42,430  LSEHS: n=106,915 |  |  | |
| Patients who had at least one record in the database more than six months prior to the index date ^‡^ | EHI: n=15,180  NHI: n=22,149  LSEHS: n=49,117 |  |  | |
|  |  |  |  | |
|  |  | Patients excluded in analysis ^†^ | EHI: n=1,919 (8.0%)  NHI: n=3,191 (7.5%)  LSEHS: n=5,897 (5.5%) | |
|  |  | Patients with diagnosis of any cancer within two years prior to the index date | EHI: n=1,919  NHI: n=3,191  LSEHS: n=5,897 | |
|  |  |  |  | |
| Full analysis set ^†^ | EHI: n=6,340 (26.5%)  NHI: n=10,857 (25.6%)  LSEHS: n=13,039 (12.2%) |  |  | |
| ^†^ Denominator is number of patients in All patients. | | | |  |
| ^‡^ Patients are duplicately counted among criteria. | | | |  |
